# Precipitation Drives the Abundance and Distribution of *Arctia virginalis*: A 40-Year Study

**DOI:** 10.1093/biosci/biaf203

**Published:** 2026-01-15

**Authors:** Richard Karban, Adam A Pepi, Patrick Grof-Tisza, Vincent S Pan, Gregory Loeb, Mikaela Huntzinger, Marcel Holyoak

**Affiliations:** Dept. Entomology & Nematology, UC Davis, Davis CA; Maine Dept of Environmental Protection, Augusta, ME; Dept. Nat. Sci., Converse Univ., Spartansburg, SC; Dept. Integr. Biol, Kellogg Biol Stn., Hickory Corners, MI; Dept. Entomology, Cornell Univ., Geneva, NY; Dept. Entomology & Nematology, UC Davis, Davis CA; Dept. Env. Sci. & Pol., UC Davis, Davis, CA

**Keywords:** abundance, distribution, insect decline, limiting factors, time series

## Abstract

To understand processes that govern the abundance and distribution of species, ecologists typically collect either long time series without surveying potential drivers or perform short-term experiments that may not scale up. We characterized the annual population dynamics of *Arctia virginalis* for 40 years and conducted experiments to examine the relative roles of abiotic conditions, host plants, predation, parasitoids, and viral infection. Rather than finding a single limiting factor, these factors were all important at some times or places. Annual densities varied by a thousand times and showed evidence of a regime shift around 2002, coincident with changing precipitation patterns. Wet sites and wet years supported higher densities, and precipitation interacted with most of the factors considered. Population control was context dependent, but water availability was generally the relevant context. Precipitation seems to be important for other Lepidoptera in western North America. Studies that include experimental tests of population drivers are required to manage insect populations.

Understanding the abundance and distribution of species is one of the most compelling goals of ecology. For example, why do caterpillars cover their host plants in some years and can barely be found in other years? Why are they abundant at one site but missing from similar sites nearby? Over the past century or so, ecologists have identified factors such as climate, resource availability (so-called bottom-up factors), and predators and diseases (top-down factors) as important influences on population sizes. However, we still lack a comprehensive understanding of the relative importance of these potential drivers for any species that interacts with many other community members.

Two main approaches have been developed to understand insect population dynamics (Hunter 2001). One involves retrospective time series of multiple species over decadal time scales. A second approach involves detailed local studies of individual species over shorter time frames. Retrospective time series of insect populations are relatively uncommon. However, studies of butterflies and moths from Europe and North America provide convincing evidence for population trends (e.g., that previously abundant species are becoming rare), but they lack detailed considerations of natural history and of the ecological mechanisms that drive the trends. Except for some studies of agriculturally or medically important species, they are rarely coupled with manipulative experiments that can strengthen inferences about the ecological drivers that govern population dynamics. At the other extreme, detailed demographic studies of focal species provide important information about factors that affect populations, but they generally include only short-term snapshots over a limited geographic range. These detailed studies may or may not scale up to explain the population dynamics observed over longer time frames.

Ecologists have developed several paradigms to explain why populations do not continue to grow larger indefinitely. Classically, plant ecologists argued that populations were not limited by the total amount of all available resources but rather by the quantity of the single scarcest resource, which then becomes the limiting factor (Liebig’s law of the minimum Liebig 1840; Tilman’s R* rule, Tilman 1982, van der Ploog et al. 1999). This hypothesis has been applied to insect populations and led to a debate between proponents of population control by predators and parasites (top-down control) versus control by limited or defended resources such as nutrients or water (bottom-up control; Hunter and Price 1992). Most current ecologists recognize that natural systems are more complex and that multiple factors may interact and colimit insect populations (Hunter and Price 1992). Furthermore, the importance of these limiting factors varies across the landscape (Gripenberg and Roslin 2007). For example, many different factors such as climate change, habitat loss, competition from invasive species, increased rates of predation and disease seem to be driving the widespread insect declines that have been observed over the past few decades (Wagner et al. 2021).

A related line of reasoning was put forward by theorists who argued that factors that regulate a population must respond to density (e.g., Sinclair 1989). They defined regulation as the ability to reduce populations when they were large and allow them to increase when small; only those sources of mortality (or reproduction or dispersal) that became more potent when densities were high and less potent when densities were low had this ability (Turchin 2003). For example, predators might concentrate their efforts in dense patches of prey and hunt less in low-density patches. However, some empiricists failed to find evidence for density-dependent regulation and argued that abiotic factors (such as climate) that were not affected by density could explain most of the variation in population sizes (Andrewartha and Birch 1954). This debate led to a consensus that only density-dependent factors can regulate populations but that other factors may affect populations more strongly much of the time.

Spatial dynamics offer an alternative to local population regulation and persistence. Some species may persist as metapopulations where local population extinctions and colonizations are in balance (Levin 1970), and other populations may not persist over the long term but may be on their way to regional extinction (Harrison 1991). These models recognize that persistence depends on dispersal among habitat patches. The majority of metapopulation studies are only a few years long, and few exceed a decade.

In summary, despite this long history of interest in population control, ecologists have an incomplete understanding of the forces that govern the abundance and distribution of insect species. Furthermore, there are relatively few studies that provide time series of insect populations. Those that do rarely include in-depth analyses to evaluate the factors that drive dynamics, both density-dependent regulators and density-independent factors that affect population sizes.

These issues have taken on a new urgency in the context of global change. For example, during the past 30–50 years, many insect populations have declined, have experienced range shifts and novel interactions, or have even gone extinct (Dirzo et al. 2014, Wagner 2020). The most complete insect data come from populations of butterflies and moths, particularly in northern Europe and North America (Boyle et al. 2019, Bell et al. 2020, Forister et al. 2021, Wagner et al. 2021, Warren et al. 2021, Edwards et al. 2025). For example, two thirds of the macromoth species in the United Kingdom have declined since 1970 (Conrad et al. 2004, Fox et al. 2014). In a well-replicated study across 63 sites in Germany, biomass of flying insect declined by 75% over the past 30 years (Hallmann et al. 2017). Many of the declines have involved previously abundant species and many of the examples have been observed within reserves or other protected areas.

Insect declines matter because insects provide the main link between plants and higher trophic levels (Harvey et al. 2023). These higher trophic levels include humans, and many of the foods we eat depend directly or indirectly on insects as beneficial organisms. Insects can also negatively affect human well-being, competing with us for crops, and serving as vectors of diseases. As a result, it is essential to understand the key factors that control insect populations.

This study is focused on the population dynamics of caterpillars of a moth, *Arctia virginalis* (wooly bears, family Erebidae, formerly *Platyprepia virginalis* [Arctiidae]) over 40 years along the coast in northern California at one protected location, the UC Bodega Marine Reserve, (38.32, –123.07). We expanded the spatial scope by examining populations at 12 locations (subsites) across the reserve over 15 years, and at 20 sites along the 1000-kilometer latitudinal range of this species over 5 years. Our approach combines an annual survey at our primary field site at Bodega Bay with observations and experiments to evaluate numerous potential drivers of population dynamics. Rather than focusing on a particular species interaction or trophic level, we have attempted to identify and evaluate many possible drivers in this one system. We have characterized the relative roles of climate, wetland habitat, host-plant abundance and quality, competitors, predators, parasitoids, and pathogens to ask what factors control *A. virginalis* over time and space.

## Natural history of *Arctia virginalis*

**Figure 1.**
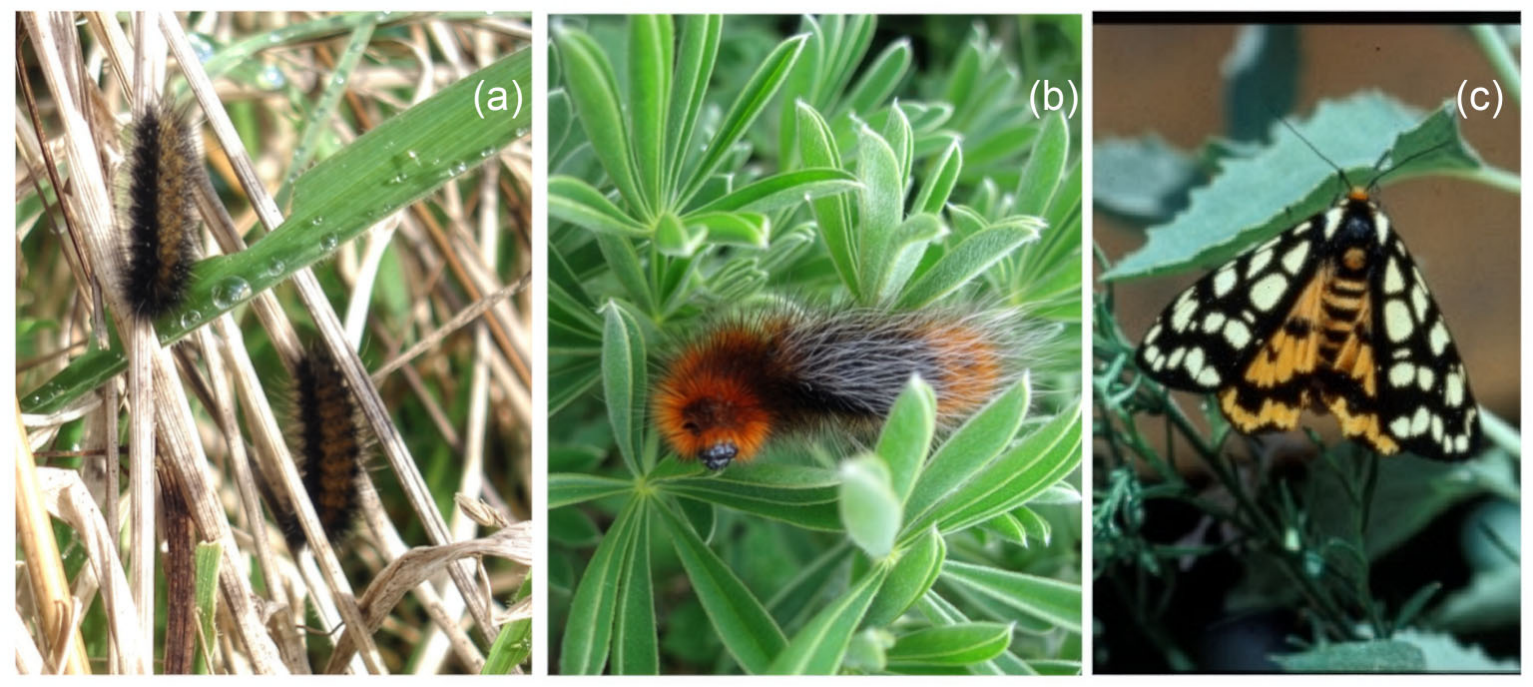
Life stages of *Arctia virginalis*. (a) Early instar caterpillars in the litter. (b) Late instar caterpillar on *Lupinus arboreus*. (c) Adult tiger moth.

*A. virginalis* has a widespread distribution throughout the western United States although populations are extremely patchy; some are separated by tens or hundreds of kilometers (Powell and Opler 2009). These moths are univoltine; females place eggs on vegetation or scatter them on the ground in late spring. Caterpillars hatch after 1–2 weeks and feed on low vegetation or litter on the soil surface through the summer, autumn, and winter. Early instar caterpillars are inconspicuous (figure 1a) and spend most of their time in the litter, moving only short distances (less than 1 meter). Like other members of the Arctiinae subfamily of Erebidae, *A. virginalis* caterpillars and ovipositing adult moths are partial to plants that contain alkaloids (English-Loeb et al. 1993, Hartmann 2009), although individuals often consume plants of many species over their lives (Singer and Bernays 2009, Karban et al. 2010). Unlike many arctiids, they do not sequester alkaloids in their tissues (Bowers 2009, Karban et al. 2010). Later instars climb up onto vegetation to feed and are conspicuous with orange–black–orange coloration (figure 1b). Last instar caterpillars typically wander many meters, likely looking for protected places to pupate (Grof-Tisza et al. 2015). Prepupae spin a silken cocoon and metamorphose into brightly colored tiger moths in late spring (figure 1c). Adults do not feed and are attracted to mating aggregations on local hilltops (Grof-Tisza et al. 2017a, 2017b). Females leave these mating aggregations to oviposit.

**Figure 2.**
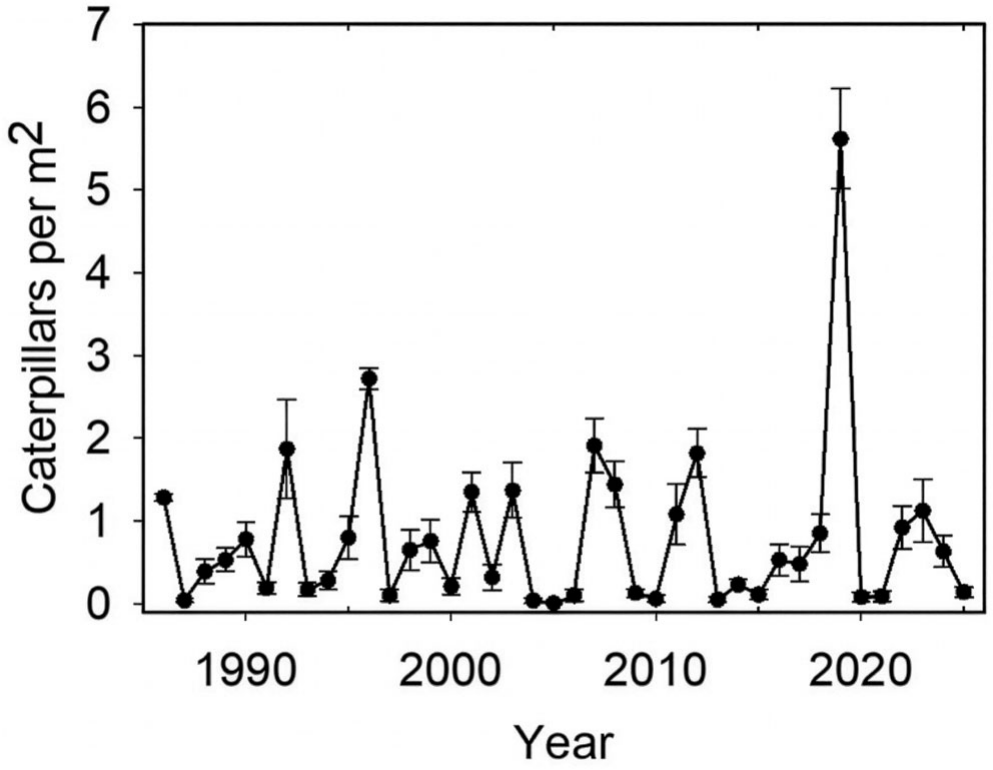
The population density of caterpillars from 1986 to 2025. Mean density was estimated as the number of caterpillars per square meter on lupine bushes east of the Bodega Marine Lab; the error bars represent the standard error. More details about the study site and survey methods are provided in Karban and De Valpine (2010).

We were initially drawn to this study system because we observed some years when caterpillars were in high abundance on almost all bushes and other years when we failed to see any. We have estimated the abundance of *A. virginalis* caterpillars (wooly bears) at our study site every year since 1985 during the last week in March. During our surveys, caterpillars were conspicuous late instars. Over the 40 years for which we have monitored caterpillar densities on lupine bushes (*Lupinus arboreus*), we have observed more than a thousand fold variation in abundance (figure 2). Explaining the high level of temporal variation in this natural population in a relatively undisturbed environment has been one of the main motivations of our work.

## Effects of abiotic factors

Early ecologists found that climate had strong effects on the abundance and distribution of animals (Shelford 1911, Uvarov 1931, Andrewartha and Birch 1954) even if these density-independent abiotic factors were unlikely to regulate populations (*sensu* Sinclair 1989). Consistent with this view, *A. virginalis* caterpillars were more abundant during spring seasons that followed wet winters compared with drier seasons. In particular, the number of large rainfall events (more than 5 centimeters of rain over 24 hours) explained 21% of the variation in caterpillar density (Karban et al. 2017). The relationship between rainfall and caterpillar numbers could be caused by many potential ecological mechanisms; more water could produce a greater quantity or quality of plant growth for some critical stage in the development of *A. virginalis*, or it could reduce the number or effectiveness of potential enemies (see the sections below). In addition, precipitation during the preceding winter and spring was associated with higher belowground moisture around lupine bushes in summer, which likely affects soil microbes (Preisser and Strong 2004), although we have not explored this pathway.

This relationship between abundant water and caterpillars was also observed across space—at Bodega Bay, caterpillars were found occurring in a wide diversity of environments but were only common in wet areas. Over the course of years, caterpillars were consistently observed in wetland sites, whereas dry upland sites were 7x more likely to experience local extinctions and recolonizations (Karban et al. 2012a, Grof-Tisza et al. 2019).

**Figure 3.**
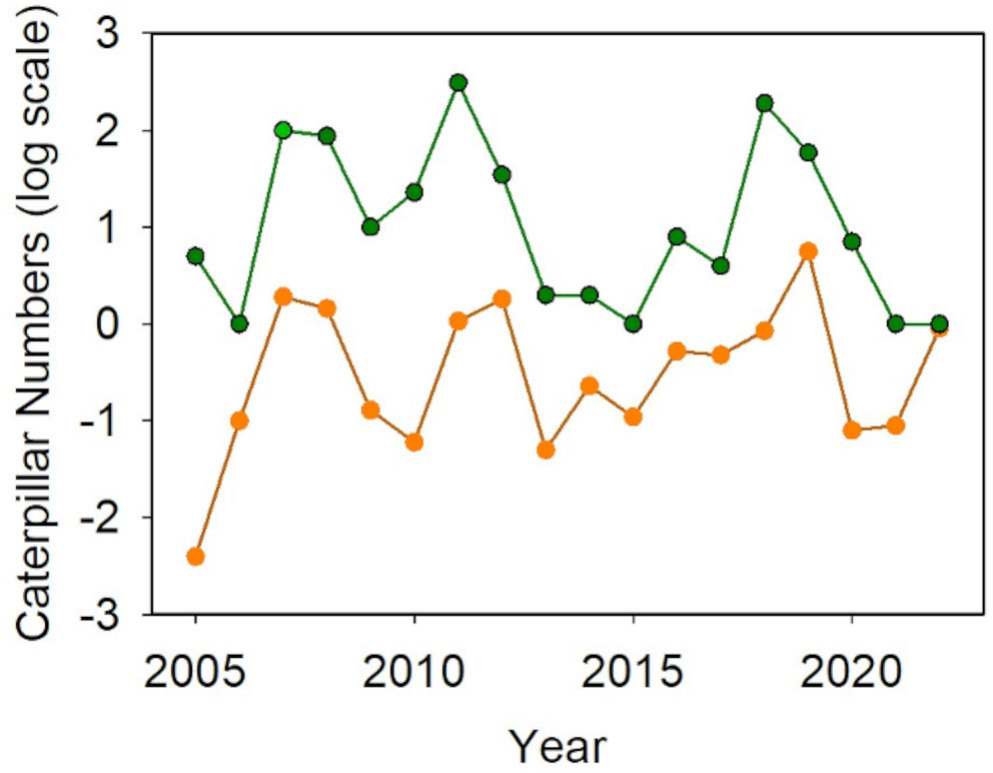
Synchronized population dynamics between our coastal site at Bodega Bay (orange, lower line) and a Sierran site near Loyalton (green, upper line), 230 kilometers to the northeast and 1480 meters higher. Both population estimates were log transformed. The estimates from Bodega were per square meter, wheareas the Sierran estimates were the number of caterpillars observed along a transect and it was not possible to express them as density per square meter.

Observations at other sites throughout the western United States were consistent with our results from Bodega Bay. For example, persistent populations in the Sierras (e.g., 39.73, –120.31), the Trinity Alps (e.g., 40.81, –122.89), and coastal Washington (e.g., 47.08, –122.71) were all located along riparian corridors or floodplains (personal observations). We have estimated the population size for caterpillars at this Sierran site every spring from 2005–2022. This site is a freshwater marsh at an elevation of 1480 meters (the green point in figure 5), approximately 290 kilometers northeast of our primary study site at Bodega Bay (the yellow point in figure 5). Caterpillars at the Sierran site use different host plant species than those at Bodega Bay. Despite the climatic and ecological differences between these two sites, caterpillar population dynamics were synchronized with those at Bodega Bay (figure 3; *r* = .57, *p* = .01 for log-transformed population estimates). Synchrony between two sites separated by this distance may be driven by correlated patterns of precipitation and water relations (the Moran effect; Moran 1953, Koenig 2002, Reuman et al. 2025). Accumulated annual precipitation at a weather station near our Sierran site was highly correlated with totals at Bodega Bay (2008–2025, *r* = .78) and at a nearby coastal site, Fort Ross, for which a longer data set was available (1985–2025, *r* = .77; see the supplemental material).

In addition to these observations, we have attempted to experimentally make dry patches more favorable for caterpillars by watering them; these manipulations were met with mixed success. Providing additional water to lupine bushes over the summer increased levels of leaf damage from insects but failed to increase survival of caterpillars (Pepi and Karban 2021). A similar watering treatment to another host plant, silverweed cinquefoil (*Argentina anserina*, formerly *Potentilla anserina*; Rosaceae), increased survival of early instar caterpillars by approximately 33% (Pepi et al. 2023). These results suggest that water may contribute to limiting caterpillars under some but not all circumstances.

Wavelet analysis that tests for the presence of oscillations of different periodicities over the length of the time series in figure 2 suggested that the population dynamics had changed during the 40 years of this study (Pepi et al. 2021). Prior to 2002 or 2003, population dynamics were characterized by short oscillations (2–3 years between peaks), but after this tipping point, longer oscillations were observed (4–6 years between peaks). This change was associated with a change in precipitation that began around 1999 when offshore sea temperatures and other climate regime indicators switched abruptly (Peterson and Schwing 2003, Cloern et al. 2010). Coincident with changes in these large-scale circulation patterns, upwelling intensity, southerly offshore flows, and marine primary productivity increased along with large changes in the biology of marine and estuarine systems (Cloern et al. 2007). Potential causal links between these climatic regimes and terrestrial populations are unclear. However, since this climatic regime shift, the positive effects of precipitation on caterpillar numbers have gotten stronger (Pepi et al. 2021).

Temperature had smaller effects than precipitation on caterpillar abundance in our surveys (Karban and De Valpine 2010). Warmer temperatures increased food consumption and growth rates of caterpillars but also made predators more effective at catching young caterpillars (Karban et al. 2015). As such, warmer temperatures may have a net negative effect on caterpillar survival (Pepi et al. 2018, Pepi and Karban 2021; see the "Interactions with predators and parasitoids" section below).

## Species interactions

**Figure 4.**
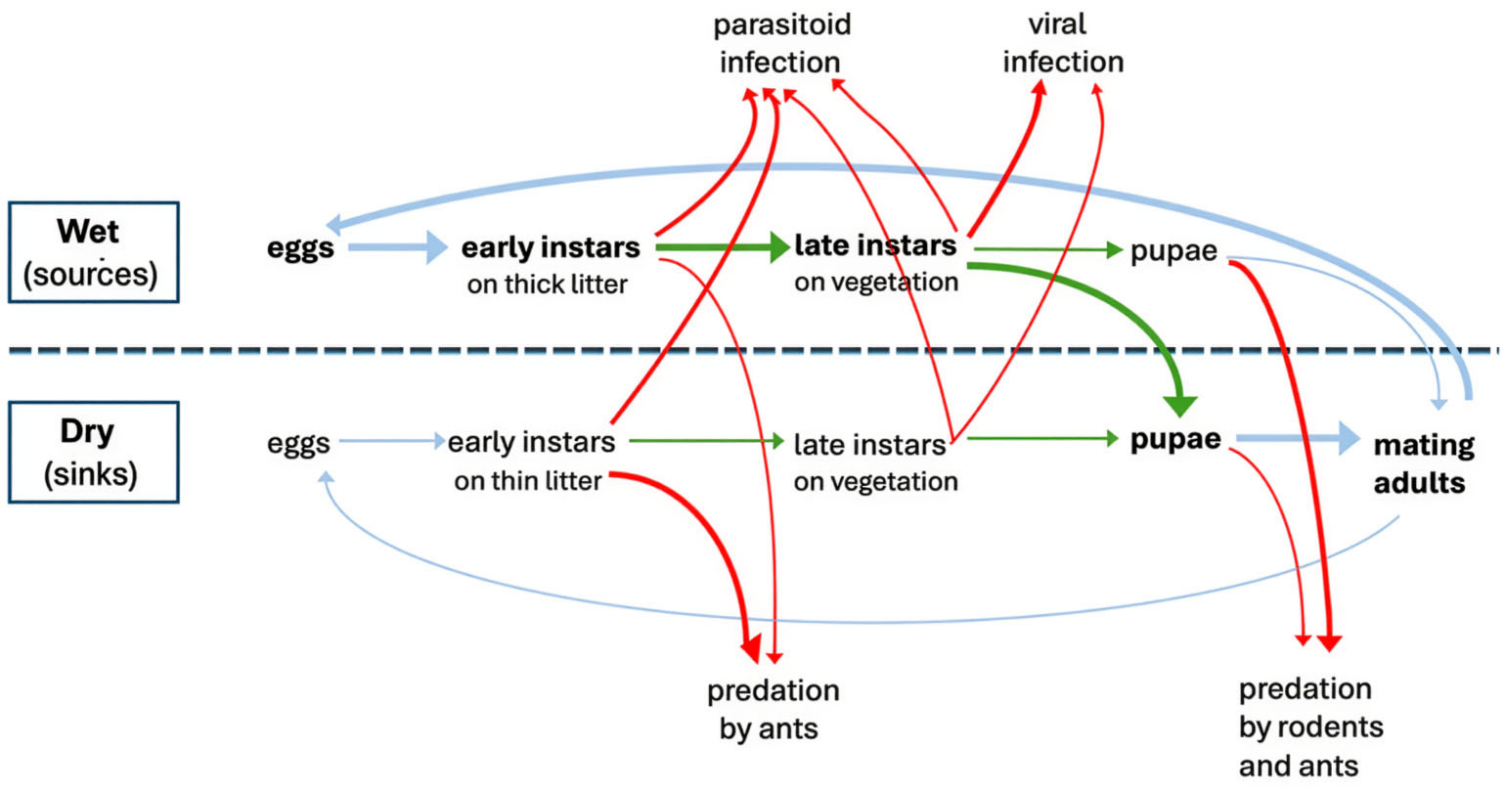
A schematic diagram of the life cycle of *A. virginalis* showing transitions between life stages in wet and dry environments. The width of lines and font sizes show the relative numbers of individuals at each stage and transition. The red lines indicate sources of mortality due to predators and parasites. The green lines indicate transitions affected by the quantity and quality of host plants. Blue lines indicate transitions not directly affected by host plants. Larval survival was low at dry sites although many late instars moved to mating sites before pupation and these were located on dry hilltops.

One approach to understanding population dynamics of a focal species is to consider the interaction web in which the species is embedded. What are the species that it eats, competes with, and risks getting eaten by? Not surprisingly, we found that different life stages of *A. virginalis* used different resources and were consumed by different enemies at different rates (summarized in figure 4).

## Effects of host plant abundance and quality

At Bodega Bay, *A. virginalis* caterpillars were strongly associated with bush lupine, which is the most abundant shrub in this coastal prairie environment (Barbour et al. 1973). Bush lupine is native to California, and Bodega Bay is near the northern limit of its distribution (Hickman 1993). It is a fast-growing nitrogen fixer that strongly affects the surrounding plant community by adding nitrogen to the soil (Maron and Connors 1996). Early instars are often found either on or beneath lupine bushes. Later instars are often on lupine but are highly polyphagous, commonly feeding on forbs and grasses (English-Loeb et al. 1993, Karban et al. 2010). Even at peak densities, *A. virginalis* caterpillars do not completely consume all of the foliage on any bushes. We have estimated the densities of both caterpillars and *L. arboreus* bushes at 12 locations on the reserve. At wet locations, which host most caterpillars, we found no relationship between lupine cover and the density of caterpillars (Karban et al. 2012b). However, at dry upland locations, where lupine is less common, we found a strong positive relationship between the density of caterpillars and the percentage of lupine cover. This suggests that the supply of lupine leaves and litter might be a limiting resource at some places. Furthermore, drier upland locations act as population sinks that go extinct every few years and are recolonized by moths that developed in other, presumably, wetter areas (Karban et al. 2012b, Grof-Tisza et al. 2019). As such, these upland locations contribute little to the population dynamics at the scale of the entire reserve in most years.

**Figure 5.**
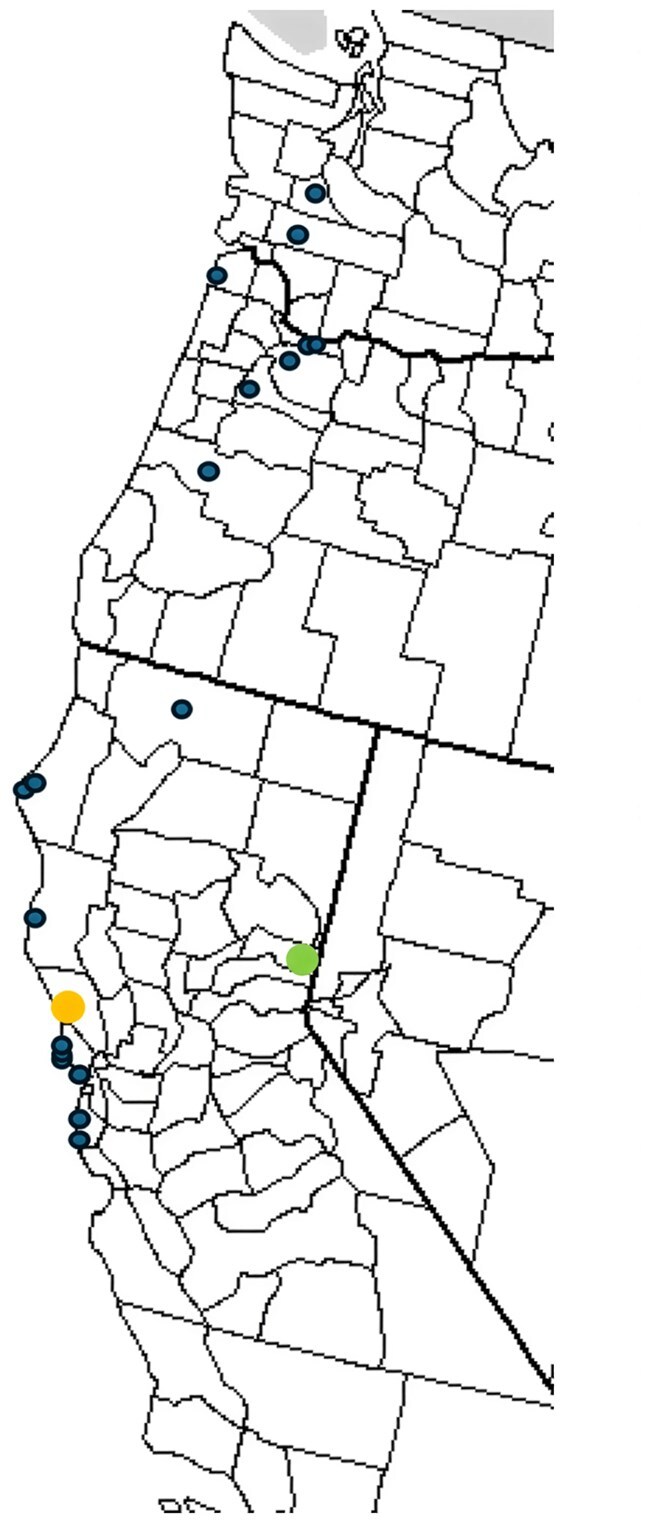
A map of the western United States showing the location of the sites that were surveyed for caterpillars. Our primary field site, Bodega Marine Reserve, is indicated with a larger yellow point at the coast. A site in the Sierras is indicated by a arger green point inland near the border with Nevada.

Stable populations are found at inland locations that lack bush lupine, clearly indicating that other host plants sustain this species. Evidence from other sites indicates that host-plant abundance may affect caterpillar numbers. A study that examined 20 populations (figure 5) over five seasons from Ano Nuevo, in central California (latitude 37.16 degrees north), to West Rocky Prairie in Washington (latitude 46.89 degrees north) revealed a positive relationship between the total biomass of host plants that are used by *A. virginalis* caterpillars and the density of caterpillars. Host-plant biomass was, in turn, strongly correlated with precipitation, particularly at drier sites.

**Figure 6.**
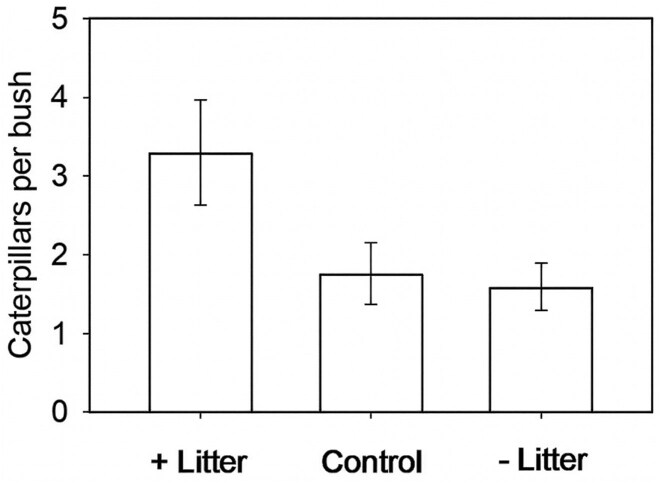
The mean number of caterpillars on bushes beneath which litter had been added (+) during the previous summer, removed (–), or unmanipulated controls. The error bars represent the standard error.

Several indirect lines of evidence suggest that food quality could limit populations of *A. virginalis*. Recall that early instar caterpillars feed on low-growing vegetation and plant litter. Access to high-quality food may be rare during the dry summers and autumns on the California coast. We experimentally manipulated the litter beneath lupine bushes by removing existing litter and low-growing vegetation (forbs and grasses in the understory) from some bushes during August and added this litter beneath other bushes (Karban et al. 2012b). By the following spring, we found more than twice as many caterpillars living on or under bushes with added litter as on or under bushes with litter removed (figure 6). This result could have been caused by litter providing more high-quality food (considered next), a moister microenvironment that reduced desiccation, or better protection from enemies.

The strong spatial and temporal relationship between precipitation and caterpillar numbers may be mediated by food quality, as was mentioned above. Lupine bushes in wet patches were 60% more likely to support caterpillars than lupine in dry patches (Grof-Tisza et al. 2019). Early instar caterpillars that were caged on lupine foliage in wet patches were more likely to survive than those caged on lupine foliage in dry patches; the mesh cages used in this experiment excluded predators and parasitoids, so these enemies were not responsible for the effect. Experimental watering of silverweed cinquefoil increased both leaf water content by 20% and survival of early instar caterpillars that were bagged on the foliage by 33% (Pepi and Karban 2021, Pepi et al. 2023).

## Interactions with other herbivore species

*A. virginalis* co-occurs at our field site at Bodega Bay with several other insect and mammalian herbivores, all of which exhibit eruptive population dynamics at times—tussock moth caterpillars (*Orgyia vetusta*), California voles (*Microtus californicus*), brush rabbits (*Sylvilagus bachmani*), black-tailed jackrabbits (*Lepus californicus*), and mule deer (*Odocoileus hemionus*).

Tussock moth caterpillars are specialists on bush lupine at our field site. Remarkably, they achieve densities that exceed those of *A. virginalis* and completely defoliate bushes during outbreaks (Harrison 1997). Tussock moth caterpillars do most of their feeding in late spring and early summer, after *A. virginalis* has already pupated. Feeding by *A. virginalis* caterpillars in early spring reduced the growth, pupal weights, and number of eggs of tussock moths that occurred on the same bushes later in the season (Harrison and Karban 1986). However, experimentally adding tussock caterpillars to bushes in summer increased the number of *A. virginalis* that developed on those bushes during the next spring relative to control bushes without additional tussock caterpillars (Karban et al. 2012a). Tussock moth caterpillars are very messy eaters and their feeding results in the accumulation of green litter beneath their host bushes. The accumulation of this deep litter provides food and favorable microhabitats for early instar *A. virginalis* caterpillars that live beneath the canopy of bushes attacked by tussock caterpillars, likely accounting for this facilitation.

Voles exhibit cyclic population dynamics in coastal grasslands in California, and as feeders on seeds and seedlings, they have significant impacts on grassland vegetation (Krebs 1966, Batzli and Pitelka 1970). John Maron established 48 experimental plots (9 × 9 meters) in dry upland areas at Bodega Bay, half of which excluded voles and other small mammals (Maron and Kauffman 2006). Voles reduced lupine establishment, particularly in years when voles were abundant (Maron and Kauffman 2006). We found strong positive correlations between lupine cover and caterpillar abundance in these plots (*r* values that ranged from .53 to .87; Karban et al. 2012b). These results suggest that voles can have negative indirect effects on caterpillar numbers that are mediated through reductions in food and shelter available to early instar caterpillars.

A separate set of 20 fenced plots was constructed by Hall Cushman to exclude larger mammals at Bodega Bay. Each 6 × 6 meter plot excluded jackrabbits, deer, both, or neither (Huntzinger et al. 2008). Eleven years after the establishment of these treatments, the plots with jackrabbits had 36% fewer caterpillars than those they could not access, whereas the plots without deer had 31% more caterpillars. Our best explanation for these effects again involved differences in host-plant abundance; several years of high jackrabbit densities reduced the cover of shrubs, forbs, and grasses in plots that the jackrabbits could access.

## Interactions with predators and parasitoids

Caterpillars at Bodega Bay are attacked by tachinid fly parasitoids (*Thelaira americana*, formerly classified as *Thelaira bryanti*). By definition, parasitoids live in or on a single host individual and kill their host during their development (Mills 2009). However, *T. americana* killed its *A. virginalis* host only approximately half of the time (English-Loeb et al. 1990). When the caterpillar survived the emergence of a parasitic fly, the parasitoid reduced caterpillar development rate and weight at pupation although adult moths were reproductively viable (Karban and English-Loeb 1997). Interestingly, caterpillars changed their preference for host plants when parasitized, which increased their likelihood of surviving after their parasitoid exited (Karban and English-Loeb 1997).

**Figure 7.**
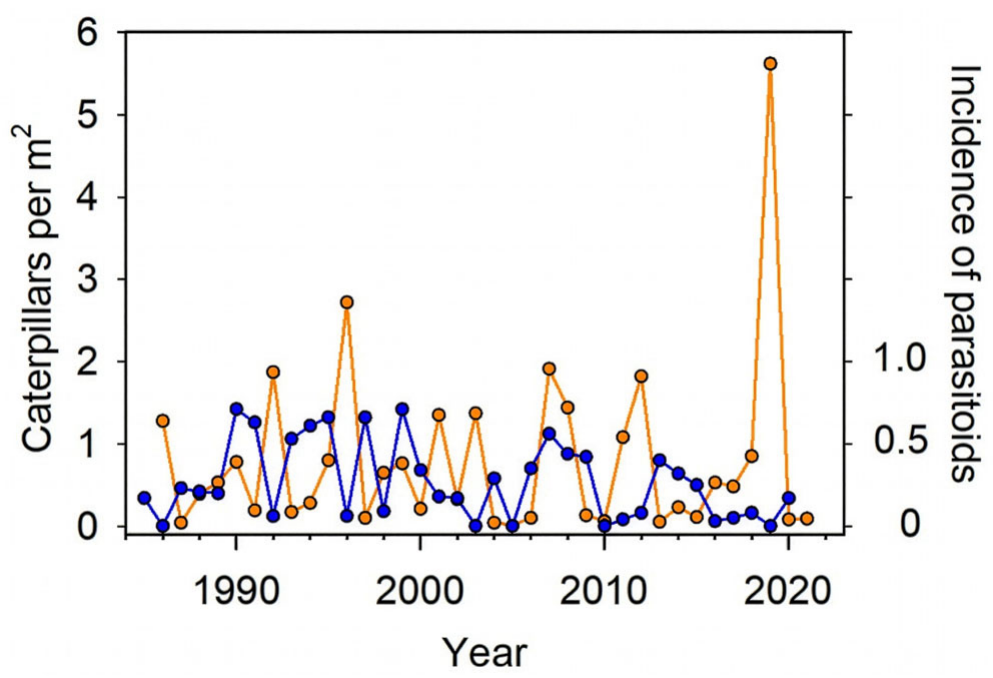
The density of caterpillars (orange 1986-2021, left axis) and the proportion of caterpillars that contained tachinid fly parasitoids (blue, 1985-2020, right axis).

The rates of infection by *T. americana* varied among years and exceeded 70% of caterpillars in some years at Bodega Bay; because parasitoids reduce the survival and reproductive capacity of moths, we suspected that they could be important density-dependent drivers of caterpillar numbers. We dissected a sample of caterpillars from one location at our field site every year from 1985 to 2020 and recorded the percentage of caterpillars that contained fly larvae (figure 7). Rather than driving changes in caterpillar numbers, the flies seemed to follow the ups and downs in the abundance of their hosts: Years with abundant caterpillars were followed by years of high rates of parasitism (Karban and De Valpine 2010).

Early instar caterpillars are also vulnerable to predation by ants; at Bodega Bay, *Formica lasioides* is the most common ant predator of *A. virginalis* (Karban et al. 2013). The workers of this ant species rip young caterpillars into small pieces that are then brought back to their nest. Caterpillars that were experimentally protected from ants were 3–5 times more likely to survive (Karban et al. 2015). Caterpillar survival was correlated with the depth of litter under their host lupine bush, and experimentally manipulating litter depth strongly affected caterpillar survival (Karban et al. 2013). Wet locations at Bodega had much deeper litter than dry locations so protection from ant predation likely contributed to the higher caterpillar densities we observed at wet areas. Differences in thermal tolerances between caterpillars and ants may play a role in this effect. Caterpillars remained active in wet litter, whereas the hunting success of ants decreased under these cooler conditions (Karban et al. 2015, Pepi et al. 2018). The number of large rainfall events during the winter (more than 4–5 centimeters of rain in 24 hours) was a good predictor of caterpillar abundance the following spring (Karban et al. 2017). Large rainfall events may drown ground-nesting predatory ants in addition to providing more litter, although we were unable to quantify the sizes of *F. lasioides* colonies. In summary, ant predation can be an important driver of caterpillar abundance and distribution, but the effect of ants depended on precipitation and the availability of litter. Ant predation of early instar caterpillars was not found to be as important across the range of *A. virginalis* as it was at Bodega Bay.

Predation of *A. virginalis* caterpillars by songbirds and other vertebrates is uncommon, presumably because the dense hairs (setae) that cover the caterpillars make them difficult for vertebrate predators to ingest (Wagner 2025). Turkeys (*Meleagris gallopavo*), which have recently become abundant at Bodega Bay and other sites in the western United States, may be an exception to this rule.

Unlike caterpillars, pupae neither move nor have hairs to protect them. Pupae were sought and consumed by mice (*Peromyscus maniculatus*) and ants. Prior to pupation, caterpillars moved away from lupine and other hosts in wet areas to prickly thistles in dry, upland areas where they were less likely to be found and eaten by predators (Grof-Tisza et al. 2015). Adult moths, despite being aposematically colored, were more commonly attacked by birds than caterpillars, although it is unclear how important bird predation is at this stage.

## Virus infection

During the past decade, we realized that eggs, caterpillars, pupae, and adults were infected with a nucleopolyhedrovirus (Pepi et al. 2022a). Most individuals contain some detectable titer of the viral occlusion bodies at Bodega Bay, as well as other sites (Pepi et al. 2022a, 2023). The side effects of the virus include poor growth, reduced activity, regurgitation of milky fluid, cloudy hemolymph, yellow fat bodies, and death. Individuals with low titer levels survived and likely transmitted the virus to their offspring. The proportion of infected individuals, infection severity, and survival to adulthood exhibited delayed density dependence (Pepi et al. 2022a). In other words, sites with higher densities of caterpillars last year exhibited a higher incidence of virus and worse outcomes in the current year. This indicates that the virus has the potential to regulate *A. virginalis* populations, although this conclusion was based on a comparison of different sites that varied in density. A time series from one site would be better for evaluating the hypothesis that the virus actually drives population dynamics of *A. virginalis*.

Abiotic conditions influenced the negative effects of the virus on survival. Infection rates were higher at wet sites, which had larger and more persistent caterpillar and viral populations (Pepi et al. 2023). Experimental watering during the dry summer had a weak positive effect on caterpillar survival, in contrast to viral infection rates. Locations with more exposure to ultraviolet radiation had lower infection rates and greater caterpillar survival (Pepi et al. 2022a). Locally higher viral loads were associated with reduced leaf damage and increased production of lupine inflorescences (Pan et al. 2023).

## Dispersal and mating behavior influence spatial distribution

We know less about factors that influence distributions over space because we have focused on patterns of abundance over time. As we mentioned earlier, populations are spatially patchy and associated with wetter environments. Upland locations were occupied inconsistently; these tended to go locally extinct and were recolonized by adults that had developed in wet microsites.

Adults fly to mating sites on ridges or hilltops when those topographic features are available (Grof-Tisza et al. 2017a, b). Individuals showed strong fidelity for hilltop locations and elevation was a better predictor of an individual’s choice of mating location than was proximity to larval patches. After mating, females disperse away from these hilltops in search of oviposition sites. The locations of mating sites likely influenced the distribution of caterpillars; larval patches farther from mating sites were less frequently colonized by ovipositing females (Pepi et al. 2022b). Population models that Included movement to and from mating sites exhibited less regional synchrony, lower rates of patch occupancy, and higher rates of colonization and extinction of larval patches.

## Lessons about population dynamics from 40 years of studying *A. virginalis*

We can now compare what we have learned from 40 years of studying this focal species with general ideas about population dynamics. Models that rely on single drivers make testable predictions and are seductively attractive because of their simplicity. However, we found evidence that water, host-plant quantity and quality, predation of young caterpillars by ants, and viral infection could all be limiting under some circumstances. As a result, we conclude that there is no single factor that regulates this species; the relative importance of different factors varied over time and space and with the availability of water.

A second take-home message from our work is that it is unrealistic to distinguish between biotic factors that are likely to be density dependent and climatic factors that are likely to be density independent. Many of the density-dependent species interactions were themselves strongly dependent on abiotic conditions. Host plants were limiting at dry locations but less so at wet sites. Ants were likely to be more effective as predators of small caterpillars when conditions were warmer and drier so caterpillars could not hide in dense litter. Viral infection rates were higher when levels of UV radiation were low. Interactions such as these completely blur the distinction between density-dependent and -independent population control; the two are so interactive as to be inseparable.

A third take-home message deals with the nature of context dependencies. The model of population dynamics that emerged from our study is far more complex and context dependent than the simple models described in the introduction, which relied on identifying a single limiting resource or on differentiating between top-down or bottom-up control. This complexity has been discouraging and has led some workers to conclude that there are no laws or generalizations possible from studies that seek to explain the abundance and distribution of species. John Lawton (1999) concluded that the “contingency becomes overwhelmingly complicated” to understand “the ecology of sets of coexisting species interacting at local scales.” He described community ecology as a mess because “almost every place, time, and species assemblage is sufficiently different to make more general patterns and rules almost impossible to find.”

We reach a much more optimistic conclusion. First, recognizing the context dependencies seems like an important first step. This recognition prevents us from accepting the simplest models. Not all insect populations show similar dynamics and different populations of the same species vary across time and space. As a result, long-term studies at multiple sites are required to accurately view population trends for any species. This is analogous to attempting to draw conclusions about long-term changes in climate from short-term weather reports. Second, identifying water availability as a significant component of the relevant ecological context takes us another step further especially in the western United States, where populations of many species are often limited by water. This finding narrows the menu of context dependencies that we must consider.

**Figure 8.**
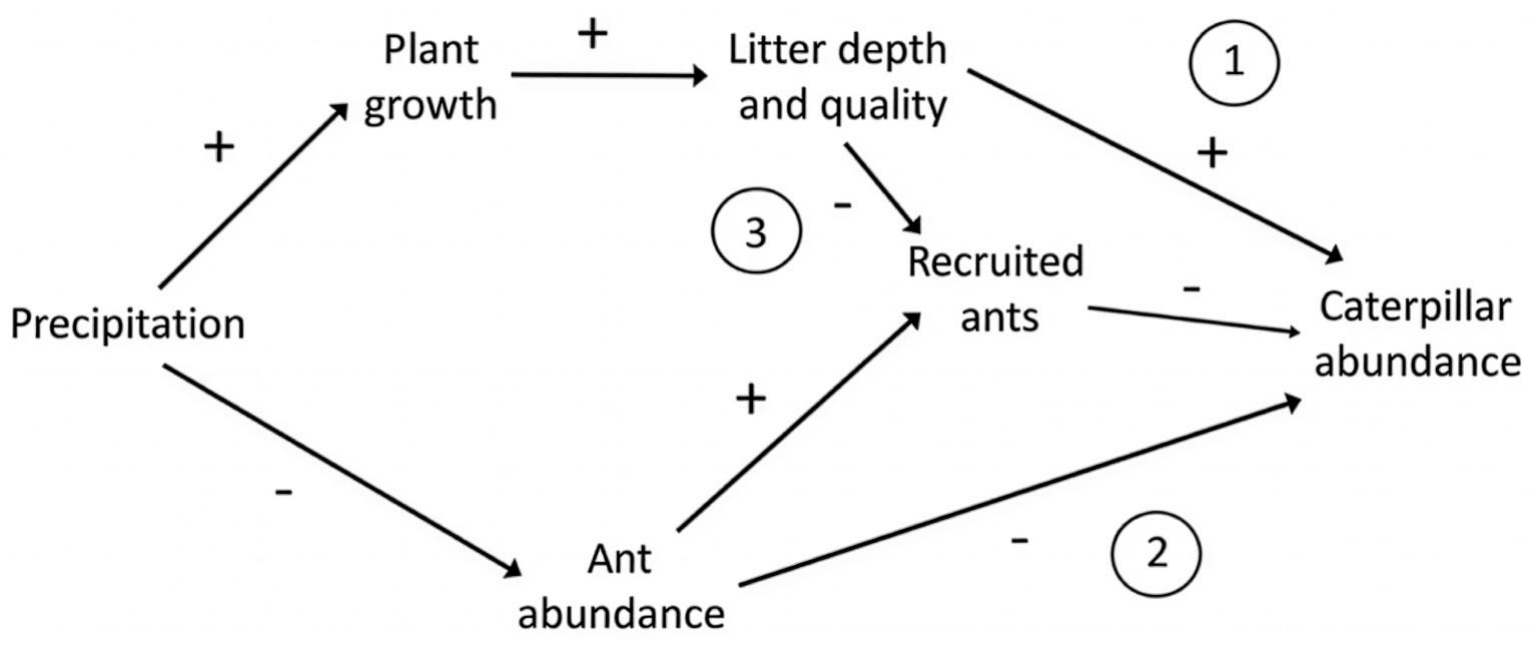
Three ecological mechanisms that link precipitation to caterpillar abundance. Mechanism 1 involves improved food quantity and quality of litter and other food (Grof-Tisza et al. 2019, Pepi and Karban 2021). Mechanism 2 involves reduced abundance of ant predators of pupae and especially of early instar caterpillars (Karban et al. 2017). Mechanism 3 involves reduced effectiveness of ant predators because of their reduced ability to discover and recruit to caterpillars in wet environments (Karban et al. 2015, 2017, Pepi et al. 2018). Other mechanisms are also possible although we have less evidence supporting these paths.

Precipitation was an important determinant of both the spatial distribution and the abundance of caterpillars over time. *A. virginalis* was strongly associated with wet sites across the landscape. Dry locations had smaller populations of caterpillars and were more likely to experience local extinction events during dry years (Karban et al. 2012b). These patterns were driven by at least three indirect ecological mechanisms (figure 8; Karban et al. 2017). Precipitation increased plant growth which provided more and higher-quality food for caterpillars (Grof-Tisza et al. 2019, Pepi and Karban 2021). Large precipitation events reduced the abundance of predatory ants which increased caterpillar survival (Karban et al. 2017). Precipitation increased litter, which made ants less successful at recruiting to and consuming caterpillars (Karban et al. 2015, 2017, Pepi et al. 2018). These local ecological mechanisms scaled up to produce larger-scale effects over time and space. Changes in the precipitation regime over the 40 years affected interactions with food and co-occurring species that were associated with changes in the population dynamics of *A. virginalis* (Pepi et al. 2021, 2023).

There are still missing pieces to this story. We have found that viral infections can respond in a density-dependent fashion, but we do not know that the virus actually drives population dynamics of this species. This will require a longer time series of both the virus and moths. Because moths are strong fliers and eggs are difficult to find, we know little about adult oviposition behavior and the factors that are important for eggs and newly hatched caterpillars. We have a detailed picture of the population at our Bodega Bay site and some evidence that moths at other locations behave similarly in many ways but differently in a few important features.

We do not know how well the lessons we have learned from *A. virginalis* will apply to other species. Many species of butterflies in western North America have experienced population declines over this time frame (Crossley et al. 2021, Forister et al. 2021, Edwards et al. 2025), a phenomenon that we did not observe in our study. These declines were loosely associated with hot and dry conditions (Forister et al. 2018, 2021, Crossley et al. 2021), although the time series described in these studies cannot, by themselves, establish whether climatic factors were the actual drivers of the declines. Other taxa that live on the west coast of North America experienced regime shifts that occurred contemporaneously with the regime shift we observed for *A. virginalis*. For example, meadow spittlebugs, *Philaenus spumarius*, were abundant and widely distributed along the California coast prior to 2001. However, their populations declined precipitously after 2002, and the species has persisted in microhabitats that provide protection from unfavorable climatic conditions (Karban and Huntzinger 2018). Farther afield, increased predation has greatly reduced populations of arctic ground squirrels (*Urocitellus parryii*) since 1998 (Donker and Krebs 2012). At roughly the same time, the peak populations of red-backed voles (*Clethrionomys rutilus*) doubled and have remained elevated since (Krebs et al. 2024). The drivers of these regime shifts are not understood.

It is useful to be aware that populations of insects are declining (or, in some cases, increasing). A detailed knowledge of these species is not required to predict their dynamics; phenomenological models, empirical dynamic models, and machine-learning approaches do a good job of predicting population changes. However, this awareness is of limited value if we do not understand the ecological mechanisms that are responsible for the changes in insect populations that we observe. A detailed understanding of the ecological drivers is required to manage populations, allowing species we value to increase and limiting those that we consider pests. For example, bringing populations of the large blue butterfly (*Phengaris arion*) back from local extinction in the United Kingdom required a detailed understanding that this species was dependent on its ability to parasitize one particular ant species (*Myrmica sabuleti*; Thomas et al. 2009). This ant required short grass habitats grazed by rabbits. This detailed knowledge of the drivers of population dynamics informed management practices and has led to a sustained recovery of this species in the United Kingdom (Thomas et al. 2025). Without this detailed knowledge of the ecological drivers, most listed species are declining even in protected areas (Dias-Silva et al. 2021, Leandro 2023). Populations of other moths and butterflies in western North America appear to respond positively to precipitation, much as *A. virginalis* does (Crossley et al. 2021). If we hope to save these and other insect species, it will be essential to learn whether similar drivers are responsible for their population dynamics.

## Supplementary Material

biaf203_Supplemental_File

## Data Availability

Data are available in Dryad: https://datadryad.org/dataset/doi:10.5061/dryad.05qfttfgs
