# Supplementary material for: Precipitation Drives the Abundance and Distribution of Arctia virginalis: A 40-Year Study
Source: Bioscience. 2026 Jan 15;76(4):375–84. doi: 10.1093/biosci/biaf203 (PMC13069562; doi:10.1093/biosci/biaf203)
Supplement: biaf203_Supplemental_File [file biaf203_supplemental_file.docx]

Precipitation Supplement

We compared annual accumulated precipitation at our sites at Bodega Bay and Loyalton. Most precipitation at both sites falls during the winter months so we considered the year as starting in October and ending the following September. We used precipitation data from NOAA Western Regional Climate Center (<https://scacis.rcc-acis.org>). Data were collected at Bodega Bay starting in 2008. To get a longer data set from the coast we also included data from Fort Ross (38.5, -123.2) from 1985-2025 although six years were missing. The closest reporting station near Loyalton on the west side of the Sierras was Downieville (39.6, -120.8) and these data were complete from 1985-2025.

| Year | Ft. Ross | Bodega | Downieville |
| --- | --- | --- | --- |
| 85 | 42.8 | . | 86.5 |
| 86 | . | . | 33.2 |
| 87 | . | . | 43.2 |
| 88 | 33 | . | 67.5 |
| 89 | 25.2 | . | 48.5 |
| 90 | 22.5 | . | 46.2 |
| 91 | 28.6 | . | 40.2 |
| 92 | 39.4 | . | 80.3 |
| 93 | 24.7 | . | 39.3 |
| 94 | 54.6 | . | 108.4 |
| 95 | 39.7 | . | 80.1 |
| 96 | 39.1 | . | 79.7 |
| 97 | 66.1 | . | 86.8 |
| 98 | . | . | 63.1 |
| 99 | . | . | 64.8 |
| 0 | 23.8 | . | 37.1 |
| 1 | 38.5 | . | 52.1 |
| 2 | 33 | . | 69.6 |
| 3 | 25.4 | . | 52.9 |
| 4 | 34.9 | . | 65.2 |
| 5 | 31.8 | . | 93.3 |
| 6 | 19.6 | . | 47.6 |
| 7 | 23.2 | . | 43.5 |
| 8 | 20.8 | 17.7 | 57 |
| 9 | 26.2 | 37.1 | 61.8 |
| 10 | 21.4 | 39 | 91 |
| 11 | 28.5 | 22.1 | 52.2 |
| 12 | . | 26.5 | 26.9 |
| 13 | 24.3 | 20.2 | 38.9 |
| 14 | . | . | 43.4 |
| 15 | 37.4 | 25.1 | 73.1 |
| 16 | 60.2 | 42.4 | 129 |
| 17 | 33.7 | 24.6 | 56.6 |
| 18 | 56.2 | 43.7 | 89.8 |
| 19 | 18.2 | 15.7 | 42.6 |
| 20 | 17.6 | 12.2 | 33.5 |
| 21 | 33.7 | 22.4 | 61.4 |
| 22 | 47.3 | 36.7 | 92.4 |
| 23 | 42.3 | 29.5 | 58.2 |
| 24 | 34.1 | 31.3 | 63.1 |
